# Incidence, time course and independent risk factors for metachronous peritoneal carcinomatosis of gastric origin – a longitudinal experience from a prospectively collected database of 1108 patients

**DOI:** 10.1186/s12885-015-1081-8

**Published:** 2015-02-19

**Authors:** Florian Seyfried, Burkhard H von Rahden, Alexander D Miras, Martin Gasser, Uwe Maeder, Volker Kunzmann, Christoph-Thomas Germer, Jörg OW Pelz, Alexander G Kerscher

**Affiliations:** 1 Department of General, Visceral, Vascular- and Pediatric Surgery, University of Wuerzburg Medical Center, Wuerzburg, Germany; 2 Division of Diabetes, Endocrinology and Metabolism, Imperial College London, London, UK; 3 Cancer Registry Mainfranken, University of Wuerzburg Medical Center, Wuerzburg, Germany; 4 Department of Internal Medicine, University of Wuerzburg Medical Center, Wuerzburg, Germany; 5 Comprehensive Cancer Center Mainfranken, University of Wuerzburg Medical Center, Wuerzburg, Germany

**Keywords:** Gastric cancer, Peritoneal carcinomatosis, Metachronous, Risk factors, Perioperative chemotherapy, Recurrence, Survival

## Abstract

**Background:**

Comprehensive evidence on the incidence, time course and independent risk factors of metachronous peritoneal carcinomatosis (metaPC) in gastric cancer patients treated with curative intent in the context of available systemic combination chemotherapies is lacking.

**Methods:**

Data from a prospectively collected single-institutional Center Cancer Registry with 1108 consecutive patients with gastric adenocarcinoma (GC), clinical, histological and survival data were analyzed for independent risk factors and prognosis with focus on the development of metaPC. Findings were then stratified to the time periods of treatment with surgery alone, 5-Fluorouracil-only and contemporary combined systemic perioperative chemotherapy strategies, respectively.

**Results:**

Despite R0 D2 gastrectomy (n = 560), 49.6% (±5.4%) of the patients were diagnosed with tumour recurrence and 15.5% (±1.8%) developed metaPC after a median time of 17.7 (15.1-20.3) months after surgery resulting in a tumour related mortality of 100% with a median survival of 3.0 months (2.1 – 4.0). Independent risk factors for the development of metaPC were serosa positive T-category, nodal positive-status, signet cell and undifferentiated gradings (G3/G4). Contemporary systemic combination chemotherapy did not improve the incidence and prognosis of metaPC (p = 0.54).

**Conclusions:**

Despite significant improvements in the overall survival for the complete cohort with gastric cancer over time, those patients with metaPC did not experience the same benefits. The lack of change in the incidence, and persistent poor prognosis of metaPC after curative surgery expose the need for further prevention and/or improved treatment options for this devastating condition.

## Background

Although the incidence and cancer-related mortality of gastric carcinoma (GC) have been decreasing steadily during the past century, it remains one of the most common malignancies and the second leading cause of cancer death worldwide [1-3]. Up to 60% of GC patients are at an advanced stage at initial diagnosis [4], with a 5-year survival rate of approximately 25% [5]. These older institution-based data have been confirmed by a recent European population-based study with 39% of patients having metastatic disease and 14% syncronous peritoneal carcinomatosis (synPC) at primary diagnosis respectively [4]. Patients with locally advanced lesions experience a high recurrence rate even after R0 resection by gastrectomy with standard D2 lymphadenectomy has been achieved [5]. Thus, different perioperative multimodal treatment regimens have been introduced during the last two decades. These vary from adjuvant chemo-radiotherapy currently preferred in the U.S. and Canada [6], a pre- or post-operative chemotherapy in Europe, postoperative chemotherapy [7,8] for 1 year in Japan, to postoperative chemotherapy with capecitabine and oxaliplatin for 6 months in Korea [8,9]. Perioperative chemotherapy has been shown to downsize and downstage gastric cancer in up to 43% of patients [10], up to the point of complete pathological response [11-13].

These developments have enabled physicians to offer patients even with locally advanced or metastatic stage at diagnosis, − a curative therapy [11-13]. Despite them, the proportion of metachronous tumour progression remains high and data on surgical and survival benefits of the perioperative chemotherapy have been controversial [10]. It has been argued that a perioperative chemotherapy induced downsizing and downstaging of the tumour may enable curative surgery with, however, initial benefits diminishing in the long term [10].

Although initially encouraging results from cytoreductive surgery and intraperitoneal chemotherapy in highly selected patients were reported, patients diagnosed with metachronous peritoneal carcinomatosis (metaPC) still face a poor prognosis [14-16]. Therefore, strategies aiming to prevent or at least delay metachronous dissemination seem to be a sound therapeutic approach in patients at high risk of recurrence [17,18].

As there has been no evidence for surveillance related survival benefit [19,20], neither the German S3 guidelines nor the ESMO-ESSO-ESTRO Clinical Practice Guidelines recommend standardized follow up. The vast majority of the data leading to this recommendation did, however, not emphasise both the current perioperative combination chemotherapeutic regimes available [21,22] and the availability of new promising treatment options [16-18].

Clarifying the relationship between clinicopathological factors, different perioperative treatment regimens and independent risk factors of recurrence can add valuable information and may lead to improved treatment and follow up programs in patients at high risk of recurrence. Here we report long-term results from the largest cohort of gastric cancer patients to our knowledge and focus on the incidence and time course of tumour progression in patients treated with curative intent, and specifically examine the role of currently available perioperative chemotherapeutic regimens.

## Methods

### Cohort definition

For this study, all consecutive patients with GC treated at the University of Wuerzburg Medical Center Cancer Registry (UWCR) between January 1986 and July 2013 were identified from the Cancer Registry of Wuerzburg University Medical Center. Patients diagnosed with other than adenocarcinoma of gastric origin or having any other carcinoma or without complete follow up were excluded. Patients were grouped into three equally long time periods ranging from 1986 to 1994 (time period I), 1995 to 2004 (time period II) and from 2005 to July 2013 (time period III) each covering profound changes in perioperative therapy, staging standards and/or diagnostic imaging available (Table 1).

**Table 1:** **Demographic and pathological tumour characteristics of 1072 patients without 30-day mortality constituting the basis for survival calculations**

Demographics and pathological tumor characteristics (n = 1108)					
**Epidemiology**					
**Patients**	**Time period I**	**Time period II**	**Time period III**	**p-value**	**All time periods**
	(1986–1994)	(1995–2003)	(2004 – 2013)		
	n = 363 (32.8%)	n = 349 (31.5%)	n = 396 (35.7%)		n = 1108 (100%)
**30d mortality**	8	14	14	0.37	36
	(2.2%)	(4.0%)	(3.5%)		(3.2%)
**Patients w/o 30d-mortality**	n = 355	n = 335	n = 382		n = 1072
	(33.1%)	(31.2%)	(35.6%)		(100%)
**Median age (y)**	63.81	65.56	65.70 y	0.13*	65.05 y
	(19.59 -91.24)	(34.33 -91.58)	(21.78 - 93.91)		(19.59 - 93.91)
**Gender m/f**	224/131 (63.1/36.9%)	216/119 (64.5/35.5%)	243/139 (63.6/36.4%)	0.93	683/389 (63.7/36.3%)
**Tumor characteristics/staging of patients w/o 30d mortality (n = 1072)**					
**UICC stage I**	82 (23.1%)	82 (24.5%)	103 (27.0%)	0.47	267 (24.9%)
**UICC stage II**	83 (23.4%)	102 (30.4%)	100 (26.2%)	0.11	285 (26.6%)
**UICC stage III**	63 (17.7%)	42 (12.5%)	57 (14.9%)	0.16	162 (15.1%)
**UICC stage IV**	111 (31.3%)	105 (31.3%)	114 (29.8%)	0.88	330 (30.8%)
**UICC stage X**	16 (4.5%)	4 (1.2%)	8 (2.1%)	0.02	28 (2.6%)
**T1/Tis**	61 (17.2%)	50 (14.9%)	61 (16.0%)	0.72	172 (16.0%)
**T2**	107 (30.1%)	141 (42.1%)	89 (23.3%)	<0.001	337 (31.4%)
**T3**	89 (25.1%)	79 (23.6%)	124 (32.5%)	0.015	292 (27.2%)
**T4**	76 (21.4%)	58 (17.3%)	92 (24.1%)	0.084	226 (21.1%)
**Tx**	22 (6.2%)	7 (2.1%)	16 (4.2%)	0.027	45 (4.2%)
**N0**	95 (6.8%)	99 (29.6%)	137 (35.9%)	0.023	331 (30.9%)
**N1**	55 (15.5%)	98 (29.3%)	132 (34.6%)	<0.001	285 (26.6%)
**≥ N2**	168 (47.3%)	119 (35.5%)	82 (21.5%)	<0.001	369 (34.4%)
**Nx**	37 (10.4%)	19 (5.7%)	31 (8.1%)	0.074	87 (8.1%)
**G1**	11 (3.1%)	5 (1.5%)	7 (1.8%)	0.302	23 (2.1%)
**G2**	79 (22.3%)	99 (29.6%)	99 (25.9%)	0.091	277 (25.8%)
**G3**	142 (40.0%)	209 (62.4%)	239 (62.6%)	<0.001	590 (55.0%)
**G4**	7 (2.0%)	1 (0.3%)	8 (2.1%)	0.093	16 (1.5%)
**Gx**	116 (32.7%)	21 (6.3%)	29 (7.6%)	<0.001	166 (15.5%)
**Signet Ring Cell**	13	8	7	0.284	28
	3,7%	2,4%	1,8%		2,6%
**synPC**	35 (9.9%)	49 (14.6%)	74 (19.4%)	0.001	158 (14.7%)
**synPC (isol.)**	11 (3.1%)	30 (9.0%)	45 (11.8%)	<0.001	86 (7.9%)
**synM+** ^(*)^	99 (27.9%)	75 (22.4%)	68 (17.8%)	0.005	242 (22.6%)
**synFM (isol.)**	75 (21.1%)	56 (16.7%)	39 (10.2%)	<0.001	170 (15.9%)
**synPC/synM+** ^(*)^	24 (6.8%)	19 (5.7%)	29 (7.6%)	0.591	72 (6.7%)

The characteristics are stratified for the three time period of treatment (*Kruskal-Wallis-Test)

Patients with synPC were diagnosed at the time of presentation with GC, either on routine staging, computed tomography or at laparotomy. Patients with metaPC were considered to be clear of peritoneal disease at the initial curative intended surgery with R0 resection, but subsequently became symptomatic on follow-up and were diagnosed with peritoneal metastases on computed tomography or at the time of another surgical exploration.

### Data source and follow up

UWCR is a central data repository maintained by the tumour registry institute of the University of Wuerzburg. It has expanded prospectively since 1985 with clinical, operative and research data of patients who were evaluated and treated at the University of Wuerzburg Medical Center. From 1985 to May 2014 it includes 146,522 patient records. Data available within the UWCR include patient demographics, histological diagnoses that are based on International Classification of Diseases coding standards (UICC Version VII, [23]), general practitioner records, inpatient admission and outpatient registration data, operating room procedures, laboratory results and computerized pharmacy records. The UWCR undergoes continuous cross platform integration with the Comprehensive Cancer Registry to ensure updated follow-up information for identification of deceased patients. Inpatient and outpatient records of all identified patients were reviewed retrospectively to extract information regarding type and duration of chemotherapy, sites of metastatic disease at presentation and disease status at last follow-up.

Patients received a symptom based follow-up according to the German S3 and the ESMO-ESSO-ESTRO Clinical Practice Guidelines [8,19]. Follow up data were obtained following contact with the family doctors on a regular basis (minimum 6 months), active collection of oncological outpatient consultation letters and pathology laboratory reports as well as automatic retrieval of patient’s live status from public registration offices (minimum once a year). The ‘Death Certificate Only’ (DCO) rate in this database is 0.2 and the completeness of follow up index is better than 0.9. For the patients in this study the follow up rate is 100%. Autopsy was not performed routinely. Demographic details of the three groups were compiled, along with clinical variables recorded at the time of primary diagnosis as well as at initial operation (tumour site and the presence of any metastases) and histological details of the resected specimen (tumour (T) category, nodal (N) category, tumour differentiation (G) and evidence of microscopic venous (V) and lymphatic vessel invasion (L)).

Metastases diagnosed within 30 days after the primary tumour were also defined as synchronous [23]. Peritoneal carcinomatosis was diagnosed usually intraoperatively and confirmed histopathologically and in other cases by computed tomography.

This study has been approved for full ethics waiver due to its retrospective nature by the University of Wurzburg ethics committee.

### Statistical analysis

The data were analyzed with SPSS, Statistical Package for Social Sciences, version 16, SPSS, Chicago, IL, USA. Clinical and histological parameters of the three groups were compared with the Mann–Whitney U or Kruskal–Wallis test for continuous data and with the w2 test for categorical variables. P < 0.05 was considered statistically significant. Univariate survival analysis was performed with the Kaplan Meier method. Cox proportional hazard modeling or “Cox regression” was used to determine predictors for the development of *metaPC* by analyzing the group patients that were tumour free after initial oncological therapy whenever univariate analysis showed any significance.

## Results

From January 1986 to July 2013 a total number of 1,372 consecutive patients with gastric cancer were identified from the Wuerzburg Medical Centre Cancer Registry (UWCR). Out of those, 1,108 patients were diagnosed with adenocarcinoma of gastric origin, without having any other carcinoma and with complete follow up. Patients without 30d-Mortality (n = 1072) were grouped into three equally long time periods ranging from 1986 to 1994 (time period I, n = 382), 1995 to 2004 (time period II, n = 335) and from 2005 to July 2013 (time period III, n = 355) each covering profound changes in perioperative therapy and staging standards as stated above.

Demographics and pathological tumour characteristics are presented in Table 1. Mean age of the entire cohort was 65.1 (Range 19.6-93.9) years with 36% female and 64% male patients. There were no significant differences in demographic characteristics such as age (p = 0.13), gender (p = 0.93), and clinical characteristics (UICC7 Stage I-IV) at the time of surgery among the patients of the three different time periods. A consistent staging of patients during time period I was not available in 4.5% of patients which was more frequent condition compared to time periods II and III (p = 0.02). Patients of time period III were more often diagnosed with locally advanced tumours (T3/T4, p < 0.001) but less frequently diagnosed with positive nodal status (p = 0.014) compared to patients of time period I and II. There were no significant differences among the particular tumour grading during time periods II and III. Of note, an accurate tumour grading was not applicable in 32.7% of the patients of time period I (p < 0.001).

Both a synPC and an isolated synPC were more frequently diagnosed in patients of time period III vs. time period I and II (p < 0.001). In contrast, a synPC metastatic state in sites other than the peritoneum, was more often diagnosed in patients of time period I and II (p = 0.005).

Overall, 95.1% of the patients received any treatment (surgery, chemotherapy), with 90.0% of the patients undergoing any type of surgery during their course of disease (palliative procedures included). Gastrectomy with D2 lymphadenectomy was performed in 60.7% (n = 605) of the patients with a significantly higher proportion in time period III compared to time period I (p = 0.001). Thereof, R0 resection was achieved in 92.6% (n = 560) overall. These parameters did not significantly differ among the patient cohorts of different time periods (Table 2). After curative resection 30- and 90-day mortality were 2.0% and 5.5% respectively with no significant differences among the different time periods (p = 0.34 and 0.46).

**Table 2:** **Overview of the therapy all patients received during the three treatment time periods**

Therapy (all patients n = 1108)					
	Time period I	Time period II	Time period III	p-value*	All time periods
	(1986–1994)	(1995–2003)	(2004 – 2013)		
**Any therapy**	341 (93.9%)	331 (94.8%)	382 (96.5%)	0.260	1054 (95.1%)
**Any operation**	332 (91.5%)	318 (91.1%)	347 (87.6%)	0.148	996 (90.0%)
**D2 gastrectomy**	178 (53.3%)	196 (61.6%)	231 (66.8%)	0.001	605 (60.7%)
**30d mortality**	3 (1.7%)	2 (1,0%)	7 (3.0%)	0.314	12 (2.0%)
**90d mortality**	8 (4.5%)	9 (4.6%)	16 (6.9%)	0.456	33 (5.5%)
**tumor-free**	157 (88.2%)	184 (93.9%)	219 (94.8%)	0.085	560 (92.6%)
**Thereof w/o 30d-mortality**	155 (98.7%)	182 (98.9%)	213 (97.3%)	0.390	550 (98.2%)
**Chemotherapy**	45 (12.4%)	60 (17.2%)	211 (53.3%)	<0.001	316 (28.5%)
**5-FU**	40 (11.0%)	45 (12.9%)	3 (0.8%)	<0.001	88 (7.9%)
Combination*	5 (1.4%)	4 (1.1%)	101 (25.5%)	<0.001	110 (9.9%)
Combination + antibody**	0 (0%)	0 (0%)	16 (4%)	<0.001	16 (1.4%)
**Chemoregimen undocumented**	0 (0%)	11 (3.2%)	91 (8.2%)	<0.001	102 (9.2%)
**Perioperative chemotherapy in curative treated and RO patients (N = 550)**					
**Patients**	155	182	213		550
**Perioperative therapy**	0 (0%)	0 (0%)	64 (30.0%)	<0.001	65 (11.6%)
**No perioperative therapy**	155 (100%)	182 (100%)	148 (70.0%)	<0.001	485 (88.4%)

Chemotherapy includes perioperative as well as second-line and palliative therapy. Information on neoadjuvant chemotherapy is provided on 550 patients after R0 gastrectomy and D2 lymphadenectomy.

*Combination of 5-FU and/or oxaliplatin, irinotecane, cisplatin, epirubicin, doxorubicin, cyclophosphamide **combination as described above + antibody therapies (Trastuzumab, Panitumumab, Catumaxumab).

Perioperative chemotherapy was applied to 12.4% (time period I) vs. 17.2% (time period II) vs. 53.2 (time period III, p < 0.001) of the patients with perioperative combination chemotherapeutic regimes being given more often in time period III compared to time period I and II (p < 0.001).

Detailed follow-up and survival data are presented in Table 3. Duration of overall follow up was 36.9 months (range 0–258). Median overall survival was 25.2% (±2.2%) with a longer median survival in patients of time period III compared to patients of time periods I and II (p = 0.37). Accordingly, the 2-, 5-, and 10-year survival rate were significantly higher in time period III compared to time periods I and II (p < 0.001).

**Table 3:** **Tumour related overall survival of the entire patient cohort without 30-day-mortality (n = 1072) and of patients that were R0 after gastrectomy and D2-lymphadenectomy, stratified for the three time periods of treatment**

Follow up of patients w/o 30d-mortality of operated patients (n = 1072)					
	Time period I	Time period II	Time period III	p-value*	
	(1986–1994)	(1995–2003)	(2004 – 2013)		
**Mean follow up time (months)**	45,3	41,5	25,0	<0.001	36.9
	(0 – 257.97)	(0–212.99)	(0–111.97)		(0 – 257.97)
**Median overall survival [months]**	21.9 (±4.36)	21.0 (±2.27)	32.4 (±5.75)	0.037	25.2 (±2.20)
**2y-survival rate [SE], n (Patients at risk)**	49.7%	47.2%	57.3%	<0.007	51.4%
	(±2.8) n = 148	(±2.8),n = 143	(±2.9) n = 148		(±1.6) n = 439
**5y-survival rate [SE], n (Patients at risk)**	36.5%	35.1%	41.6% (±3.2)	<0.043	37.8% (±1.7)
	(±2.8) n = 88	(±2.7) n = 95	n = 46		n = 229
**10y-survival rate [SE], n (Patients at risk)**	33.8% (±2.8) n = 68	29.7% (±2.7) n = 45	38.5% (±3.4) n = 50	< 0.037	33.4% (±1.7), n = 113
**Follow up after RO resection (w/o 30d mortality, n = 550)**					
**Mean follow up time [months]**	83.1 (1.02 – 257.97)	66.4 (1.02 – 212.99)	34.7 (1.0 - 111.97)		58.8 (1.02 – 257.97)
**Median overall survival (months)**	Not reached	Not reached	Not reached		Not reached
**75% survival [months]**	47.0 (±5.7)	28.0 (±5.7)	33.0 (±7.0)	0.313	34.3 (±4.1)
**2y-survival rate [SE], n (Patients at risk)**	86.9% (±2.8)	79.5% (±3.1)	83.6% (±2.9)	0.188	83.2% (±1.7)
	n = 119	n = 129	n = 124		n = 372
**5y-survival rate (SE), n (Patients at risk)**	68.7% (±4.0)	61.9% (±3.9)	68.7% (±4.0)	0.340	65.7% (±2.3)
	n = 78	n = 90	n = 42		n = 210
**10y-survival rate (SE), n (Patients at risk)**	61.9% (±4.4)	54.3% (±4.1)	--	0.270	59.0% (±2.6)
	n = 60	n = 44			n = 104
**metaPC (general)**	10.5% (±2.8)	16.1% (±3.1)	18.9% (±3.3)	0.074	15.5% (±1.8)
	n = 13	n = 23	n = 28		n = 64
**Recurrence**	49.5% (±6.7)	46.4% (±4.0)	38.2% (±4.4)	0.523	49.6% (±5.4)
	n = 59	n = 74	n = 55		n = 188

Mean duration of follow up after R0 resection was 58.8 (+/−) months. The 2-, 5-, and 10-year survival rate were 83.2%, 65.7% and 59.0% respectively. There were no significant differences among the different time periods. Patients of time periods I and II presented significantly more frequently with metachronous metastatic state if compared to patients of time period III (p = 0.01), while patients of time period III were more often diagnosed with isolated metaPC (p = 0.03).

Pathological tumour characteristics of patients who received perioperative chemotherapy were not different to those of patients who did not (p = 0.47). Neoadjuvant chemotherapy neither impacted on survival nor on the time course of tumour recurrence (Table 4).

**Table 4:** **Overview of stage, overall survival and tumour-free survival of 550 tumour-free patients after initial therapy stratified for neoadjuvant systemic therapy**

Perioperative vs. no perioperative therapy (n = 550)			
	Perioperative therapy	No perioperative therapy	P
	n = 64	n = 486	
**pUICC7 I**	24	224	0.47
	(37.5%)	(46.1%)	
**pUICC7 II**	28	197	
	(43.8%)	(40.5%)	
**pUICC7 III**	11	56	
	(17.2%)	(11.5%)	
**pUICC7 X**	1	9	
	(1.6%)	(1.9%)	
**Median overall survival (months)**	Not reached	Not reached	0.41
**75%-survival (months) (Standard error)**	28.9% (±6.1)	36%.9 (±4.4)	
**Time to recurrence (25% of patients) (Standard error)**	17.0 (±5.5) months	25.0 (±2.5) months	0.82

**Figure 1:**
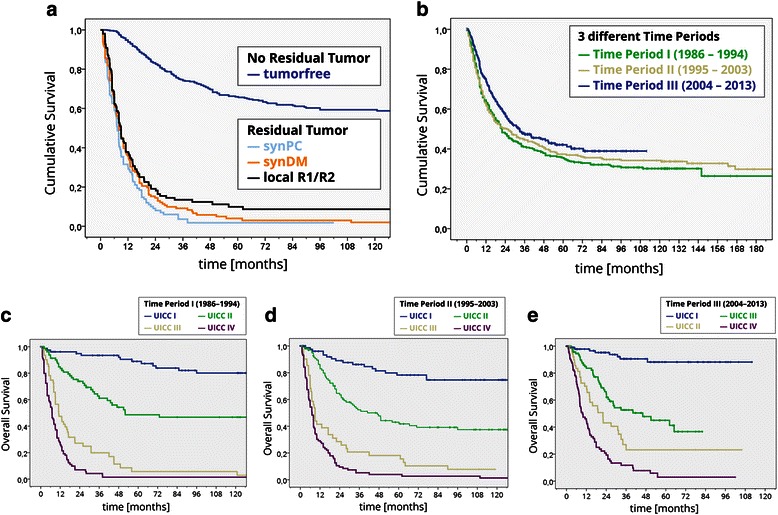
**Survival. a)** Overall survival among the different time periods of patients without 30-day mortality (n = 1072). **b)** Overall survival after R0 resection/vs. residual tumour. **c-e)** Survival among the different time periods based on UICC staging system.

Overall survival was improved in patients (n = 1072) treated in time period III (26,0 months (range 20,5 - 31,5)) compared to patients of time period I (16,0 months (range 12,5 to 19,4; p = 0.07) and compared to time period II (18.0 months (range 14,0 to 22,0; p = 0.031, Figure 1).

After R0 D2 gastrectomy (n = 550) we did not observe significant survival differences among patients treated in different time periods (time period I: 68,0 months (range 45,8 to 90,2) vs. time period II: 60,2 months (range 46,3 to 74,1) vs. time period III (65,0 months (range 39,4 to 90,6; p = 0.67).

**Figure 2:**
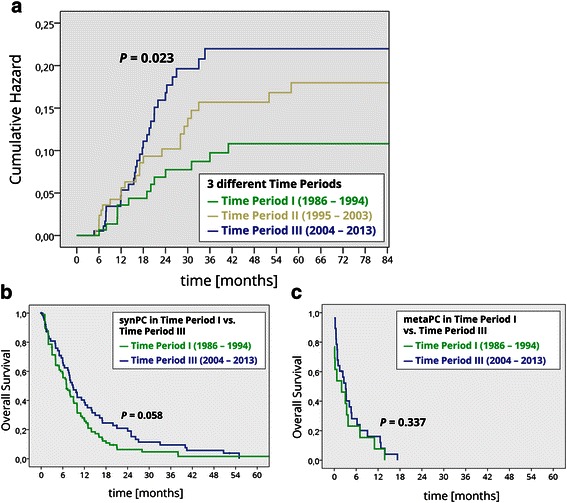
**Peritoneal carcinomatosis. a)** Cumulative hazard ratio for the development of metaPC (550 after R0 resection, stratified for the three time periods.) **b)** Tumour related overall survival of 167 patients with synchronous peritoneal carcinomatosis (synPC) stratified for time periods I and III. **c)** Tumour related overall survival from the time of diagnosis metachronous peritoneal carcinomatosis (metaPC) in the group of 550 patients that were R0 after initial therapy.

The cumulative Hazard risk for metaPC was increased for patients treated in time period III compared to those being treated in time periods II and III (p = 0.023, Figure 2a). Survival of patients diagnosed with synPC was in trend prolonged in time period III compared to time period I (p = 0.58, Figure 2b). There was no era specific survival difference in patients diagnosed with metaPC (p = 0.34, Figure 2c). The histopathological characteristics such as undifferent ‘gradings’ (G3/4, factor: 2.03 (3.65-1.13, p = 0.018), nodal positive category (N+, factor: 2,39 (4,26-1,34, p = 0.003), signet ring cell (factor: 3,88 (9,71-1,56, p = 0.004), and locally advanced tumour category (T3/4, factor: 2.35 (1.35-4.12, p = 0.003) were identified to be independent risk factors for the development of *metaPC* after R0 D2 gastrectomy (n = 550). Other factors, such as time period of treatment, age, gender, and perioperative therapy did not impact on the risk of *metaPC* in multivariate analysis (Table 5, Figure 2b).

**Table 5:** **Analysis using Cox regression model for independent risk factors after R0 D2 gastrectomy (n = 550)**

Risk factors for metachronous peritoneal carcinomatosis			
	Risk factor	P	Factor* (CI 95% confidence interval)
**Independent significant risk factors**	T3/4 (serosapositive)	0,003	2,35 (1,35 - 4,12)
	Signet ring cell	0,004	3,88 (1,56 - 9,71)
	Nodal positive	0,003	2,39 (1,34 - 4,26)
	Grading 3/4	0,018	2,03 (1,13 - 3,65)
**Non significant risk factors**	Time period (I)	0,247	1,39 (0,69 - 2,81)
	Age (<50y)	0,776	0,82 (1,65 - 0,41)
	Sex (male)	0,399	1,25 (2,08 - 0,75)
	Perioperative chemotherapy	0,392	0,68 (1,64 - 0,28)

*Is the factor by witch the risk for development of metachronous PC is increased.

## Discussion

Data from our cohort, which is to our knowledge the largest ever studied, show that neither the incidence nor the time course and prognosis of metachronous peritoneal carcinomatosis in gastric cancer patients treated with curative intent, have changed over the last three decades. Our data are in line with previous studies reporting an advanced tumour stage in 45.9 percent of the patient cohort and with synPC PC in 14.6 percent at primary diagnosis respectively [4]. Our findings show that tumour stage at diagnosis has not changed and, especially, the rates of synPC has not decreased within the last two decades, which is consistent with data reported by a recent European population based study [4]. This could firstly be explained by the fact that screening for gastric cancer is only recommended for a small proportion of people with well-established risk factors [19,24,25], and secondly, by the overall poor perception of any cancer screening programs [26].

As conclusions on synPC in gastric cancer in a single-centre study need to be drawn with caution we moved on and focused on the incidence of *metaPC* in our cohort of 1,108 patients with a median follow up of 36.9 months and a follow up rate of 100 percent.

For further analysis we excluded patients with both, a metastatic state and/or positive margins after D2 gastrectomy, as they are associated with a very poor prognosis, [27,28] and revisional surgery and/or radio chemotherapy is recommended under these circumstances [19,20]. Despite Ro D2 gastrectomy 50% of the patients were diagnosed with tumour recurrence, 16% developed metaPC after a median time of 17.7 (15.1-20.3) months from surgery resulting in a tumour related mortality of 100% with a median survival of 3 months. The data on the incidence of metaPC from this study, however, vary considerably [29-33] with that previously reported, as in our cohort peritoneal tumour progression occurred in up to 60% of patients. The differences in the results of our study cohort could be explained by several reasons. Firstly, baseline demographic and tumour characteristics of our patient cohort were different to those of other studies. Secondly, the vast majority of the previous findings were obtained from an Asian population being reported to develop gastric cancer with a more malignant tumour biology [33]. Thirdly, the patient cohorts of those studies may have had a more structured follow up postoperatively, more extensively exploiting the prevalence reserve of metachronous PC. Fourthly, older studies were published more than 13 years ago and therefore did not incorporate the currently available perioperative chemotherapeutic regimes [5,10]. For this precise reason we performed a subgroup analysis using three time periods based on the different perioperative chemotherapeutic regimes available over the last few decades (Table 2).

**Figure 3:**
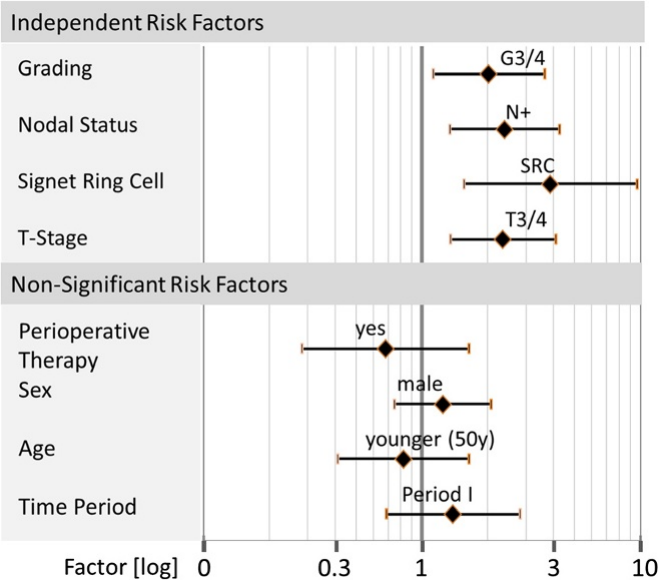
**Cox regression multivariate analysis for independent risk factors for the development of metaPC.** X-axis shows the factor by which the risk is influenced (logarithmic diagram). P-Values and risk factors are summarized in the table below the graph.

In concordance with the literature we observed an improved overall survival in patients treated with modern combination chemotherapies during time period III [5,34]. Survival after R0 D2 gastrectomy did, however, not differ in patient among the different time periods. It appears that patients diagnosed with metaPC also did not experience survival benefits as the subgroup analysis did not show significant survival differences among the three different time periods. Interestingly, patients in our cohort were more often diagnosed with metaPC and had an increased risk for metaPC during the most recent time period compared to the patients treated within the earlier time periods. However, multivariate analysis did not show that the time period of treatment itself was an independent risk factor for the incidence and prognosis of metaPC (Table 5, Figure 3).

The higher incidence of metaPC during the most recent time period may, on one hand, reflect the usage of an improved and, therefore, more sensitive imaging technology [35]. We also assumed that patients underwent imaging studies more frequently as potent second and third line chemotherapeutic regimes became available and were applied more frequently to these patients. Although pathological staging characteristics (measured by UICC VII) of the different cohorts were not different at the time point of surgery, a locally advanced tumour was found more frequently in patients in time period III. Multivariate analysis revealed that a serosa positive tumour category was an independent risk factor for the development of metaPC (Table 5, Figure 3)*,* which is in line to previous reports [36,37]. Further, it is probable that the use of perioperative chemotherapeutic regimen during the last decade may have downsized and downstaged the tumour enabling curative surgery with benefits diminishing in the long term [10]. Therefore, it could be argued that the perioperative therapy may have enabled a curative treatment in a substantial number of patients who most likely would have been treated using palliative intentions in the former time periods. It has firstly been hypothesized that preoperative chemotherapy may decrease the biological activity of the tumour and therefore reduce the likelihood of malignant biological active cell spread into the peritoneal cavity during surgery [34]. In addition, tumour dissemination during surgery through the opening of lymphatic channels lymph node dissection and spread of viable cancer cells into the peritoneal cavity during could be reduced [10]. However, we did not observe a clear benefit in our cohort.

Multivariate analysis did not identify perioperative chemotherapy, age and gender as independent risk factors for the development of metaPC (Table 5, Figure 2b). Other histopathological characteristics such as undifferentiated ‘gradings’ (G3/4), nodal positive category (N+), signet ring cell (SRC), and serosa positve tumour category (T3/4) were identified to be independent risk factors (Table 5, Figure 3), which is consistent to the literature [38,39]. Notably, median survival of patients diagnosed with metaPC was consistently shorter compared to patients diagnosed with synPC with no trend in further subgroup analysis. This may indicate that a systematic follow up of patients at high risk of tumour progression may be beneficial if strategies to treat PC become available. Despite improvements in overall survival, the time course and occurrence rate of metaPC as well as prognosis did not improve over time. Considering these disappointing results two conclusions need to be drawn: Firstly, other strategies aiming to prevent or at least relevantly delay metachronous dissemination may be a reasonable approach. A combined strategy of cytoreductive surgery and intraperitoneal chemotherapy showed favorable results in highly selected patients [16]. This multimodal treatment is currently regarded as the only therapeutic option for selected patients with PC from gastric cancer, reporting improved 5-year survival rates ranging from 13 to 28% [14,15]. Secondly, patients treated with curative intention (D2 gastrectomy plus state of the art chemotherapy) at high risk for metaPC could be tailored to other therapeutic approaches such as the extensive intraoperative peritoneal lavage (EIPL). This straightforward adjuvant surgical technique has been advocated as a useful tool for those gastric cancer patients who are likely to suffer from peritoneal recurrence [17,18]. In a randomized controlled study the effect of EIPL therapy on prevention of peritoneal recurrence on patients with peritoneal free cancer cells without overt peritoneal metastasis was verified [17,18]. Another strategy analogous to that proposed by Elias et al. could potentially apply for patients diagnosed with gastric cancer. Elias et a. scheduled patients diagnosed with colorectal cancer at high risk for metaPC to a second look laparotomy routinely even though imaging studies did not reveal any signs of recurrence at this time point [40].

## Conclusions

Despite significant improvements in the overall survival for the complete cohort with gastric cancer over time, those patients with metaPC did not experience the same benefits. Our data show that neither the incidence nor the prognosis of metachronous peritoneal carcinomatosis in gastric cancer patients treated with surgery and modern systemic chemotherapy has changed. Therefore, efforts should be made to accelerate the development and implementation of improved prevention and/or treatment options for this devastating condition. We advocate that patients at risk should be tailored to prospective trials in order to increase the evidence for promising treatment options.
